# High Efficiency, Low Power-Consumption DFB Quantum Cascade Lasers Without Lateral Regrowth

**DOI:** 10.1186/s11671-017-2064-2

**Published:** 2017-04-19

**Authors:** Zhi-Wei Jia, Li-Jun Wang, Jin-Chuan Zhang, Feng-Qi Liu, Yu-Hong Zhou, Dong-Bo Wang, Xue-Feng Jia, Ning Zhuo, Jun-Qi Liu, Shen-Qiang Zhai, Zhan-Guo Wang

**Affiliations:** 1 Key Laboratory of Semiconductor Materials Science, Institute of Semiconductors, Chinese Academy of Sciences, Key Laboratory of Low Dimensional Semiconductor Materials and Devices, Beijing, 100083 China; 2 College of Materials Science and Opto-Electronic Technology, University of Chinese Academy of Sciences, Beijing, 101408 China

**Keywords:** Quantum cascade laser, Distributed feedback, Low power-consumption, High efficiency

## Abstract

Very low power-consumption distributed feedback (DFB) quantum cascade lasers (QCLs) at the wavelength around 4.9 μm were fabricated by conventional process without lateral regrowth of InP:Fe or using sidewall grating. Benefitted from the optimized materials and low waveguide loss, very low threshold current density of 0.5 kA/cm^2^ was obtained for a device with cavity length of 2 mm. Combined with the partial-high-reflection coating, the 1-mm-long DFB QCL achieved low power-consumption continuous wave (CW) operation up to 105 °C. The CW threshold power-consumptions were 0.72 and 0.78 W at 15 and 25 °C, respectively. The maximum CW output power was over 110 mW at 15 °C and still more than 35 mW at 105 °C. At 15 °C, wall-plug efficiency of 5.5% and slope efficiency of 1.8 W/A were deduced, which were very high for low power-consumption DFB QCLs.

## Background

In recent years, quantum cascade lasers (QCLs) have proven to be very efficient coherent light sources in the mid-infrared region with a wide variety of applications, such as trace gas sensing, high-resolution spectroscopy, and free space communication [1–3]. Since the invention of QCLs [4], distributed feedback (DFB) QCLs have also attracted much attention for their single longitudinal mode operation and compact module [5–7]. In portable applications, the input electrical power consumption of the DFB QCL is required to be as low as possible, so that the total volume and power consumption of the system can be minimized. Moreover, in order to obtain stable and narrow line-width single-mode emission, continuous wave (CW) operation of DFB QCL above ambient temperature is desired. Several works on the demonstration of room temperature, low power-consumption DFB QCLs have been reported, in which narrow ridge and short cavity device structure are the common approach to lower the power consumption, with the typical ridge width of 2–5 μm [8–10]. However, the narrow ridge width will increase the waveguide loss due to the lateral optical absorption and scattering, leading to a high threshold current density (*J*_th_) and preventing the high temperature CW operation. To reduce waveguide loss of narrow ridge devices, the buried heterostructure (BH) configuration is necessary, which involves a complex fabrication process. Meanwhile, the BH structure reduces the optical confinement factor, which is also against to achieve low *J*_th_ operation. In [11], Briggs et al. used sidewall grating as an alternative to the BH structure, but the loss is still high for the narrow ridge width (4 μm). As the result, the *J*_th_ was high (1.91 kA/cm^2^ at 20 °C). In short, narrow ridge structure can lower the threshold current and power consumption at the expenses of *J*_th_, slope efficiency, and wall-plug efficiency (WPE), which is not helpful to further reduce power consumption. The complex process and high cost are also unfavorable to the fabrication and application of DFB QCLs.

In a previous report, we demonstrated the very low threshold operation of QCL in CW mode with the *J*_th_ of 0.56 kA/cm^2^ at 20 °C by optimizing growth parameter of the epitaxial materials, wide ridge width was used to lower the waveguide loss and no lateral regrowth of InP:Fe was necessary [12]. The very low *J*_th_ is encouraging for developing low power-consumption DFB QCLs without a very narrow ridge.

In this work, an active region based on two-phonon resonance design was modified and optimized. DFB QCLs with ridge width of 9 μm were fabricated by a conventional process without regrowth of InP:Fe or using sidewall grating. At 20 °C, the DFB QCLs with high-reflection (HR) coating and different cavity lengths (1, 1.5, and 2 mm) can all achieve CW single-mode operation with low *J*_th_ (0.84, 0.52, and 0.5 kA/cm^2^). In order to reduce the high mirror loss of short cavity devices, partial-high-reflection (PHR) coating consisting of Al_2_O_3_/Ge was designed and used [13]. After PHR coating on the front facet, the CW *J*_th_ of the 1-mm-long device was reduced to 0.64 and 0.69 kA/cm^2^ at 15 and 25 °C corresponding to the power consumptions of 0.72 and 0.78 W, respectively. Single-mode emission was maintained from 15 to 105 °C, and the lasing wavenumber at 150 mA was tuned from 2045.5 to 2031.2 cm^−1^; no mode hopping was observed. The maximum CW output power was over 110 mW at 15 °C and still more than 35 mW at 105 °C. Wall-plug efficiency (WPE) of 5.5% and slope efficiency of 1.8 W/A were deduced at 15 °C, which were very high for low power-consumption DFB QCLs.

## Methods

The QCL active region was based on the two-phonon resonant design, consisting of strain compensated In_0.67_Ga_0.33_As/In_0.37_Al_0.63_As superlattice, which was similar to that in ref. [14]. In order to obtain the lasing wavelength around 4.9 μm, the active region structure of ref. [14] was modified. The thicknesses of In_0.67_Ga_0.33_As quantum well layers were increased by 8%. According to our theoretical simulation on the modified active region structure, the dipole matrix element and the phonon scattering lifetime between the upper laser level and the lower laser level were 1.60 nm and 2.55 ps, respectively. The simulation results indicated that high performance can be maintained compared to ref. [14]. Moreover, the drop of the transition levels in the modified structure lead to lower electric field intensity, meaning the decrease of operating voltage.

**Fig. 1:**
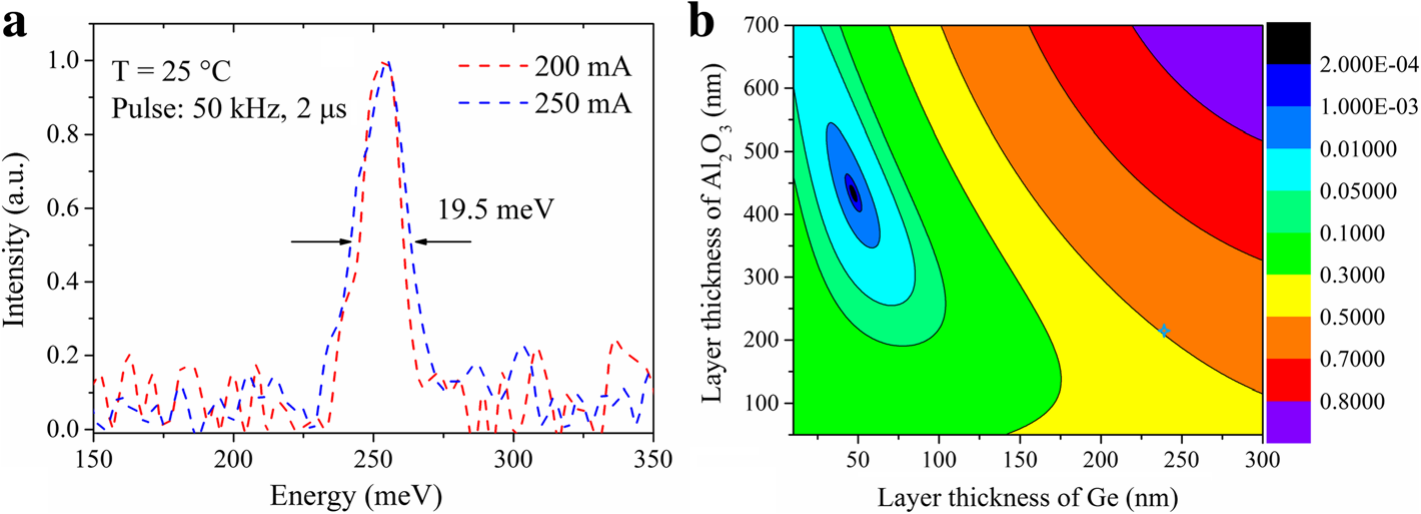
**a** Electroluminescence spectra of the wafer and **b** reflectivity of the Al_2_O_3_/Ge coating for the wavelength of 4.9 μm as a function of the thicknesses of the two layers

In order to minimize the free carrier absorption loss in the n-doped (S, 2 × 10^17^ cm^−3^) InP substrate and improve the material quality, a 3-μm-thick low-doped (Si, 2 × 10^16^ cm^−3^) InP layer was first grown on the substrate by metal organic vapor phase epitaxy (MOVPE). Then the 40-stage active region with the total thickness of 2.1 μm sandwiched between two 300-nm-thick lattice-matched InGaAs confinement layers were grown by solid-source molecular beam epitaxy (MBE) following the optimized growth control described in ref. [12]. The thicker active region can enhance the optical confinement factor and reduce *J*_th_. High interface quality was achieved as indicated by the electroluminescence (EL) spectra given in Fig. 1a, which has a narrow full-width-at-half-maximum (FWHM) of 19.5 meV. This was important for low *J*_th_ operation, because sharp interface can reduce interface scattering for both electrons and photons. The buried DFB grating was patterned on the upper InGaAs layer, followed by the growth of upper InP layers by MOCVD. The design of the DFB grating was based on finite-element method by using the commercial software COMSOL. The grating was fabricated by holographic lithography and wet etching. The period and depth of the grating were 759 and 150 nm, respectively. The upper InP layers included a 3-μm-thick low-doped (Si, 2 × 10^16^ cm^−3^) InP layer, a 200-nm-thick gradual-doped (Si, 1–3 × 10^18^ cm^−3^) InP layer, and a 500-nm-thick high-doped (Si, 8 × 10^18^ cm^−3^) InP layer. With the gradual-doped layer, a sharp drop of carrier concentration could be avoided, which was beneficial to electric injection and removing surface plasma absorption. Then the wafer was processed into the standard double-channel ridge waveguide lasers, which were simpler and low cost than the lasers with BH fabrication process. The processing details were similar to those in ref. [6]. The laser ridge width was 9 μm, and the laser chips were cleaved into different cavity lengths of 1, 1.5, and 2 mm. After HR coating on the back facet, the lasers were bonded epi-layer side down on an AlN submount with AuSn solder, wire bonded, and then soldered on a copper heat sink.

To lower the mirror loss for short cavity DFB QCL, a partial-high-reflection (PHR) coating consisting of Al_2_O_3_/Ge was deposited on the front facet of the device by electron beam evaporation. The design of Al_2_O_3_/Ge coating was based on the concept of optical admittance [15]. The reflectivity (*R*) of the Al_2_O_3_/Ge coating for the wavelength of 4.9 μm as a function of the thicknesses of the two layers is plotted in Fig. 1b. As shown, the reflectivity of the Al_2_O_3_/Ge coating can be varied from 3.85 × 10^−4^ to more than 0.9 by changing the thicknesses of the two layers. Here we chose the thickness of Al_2_O_3_ and Ge to be 220 and 240 nm, corresponding to *R* ~ 0.5, as marked by the blue star. It should be pointed out that since the waveguide loss of a specific device cannot be obtained accurately and the coating layer thicknesses cannot be controlled precisely by electron beam evaporation, the choice of *R* ~ 0.5 was not optimal, only with the purpose to lower the *J*_th_.

## Results and Discussion

The lasers were mounted on a thermoelectrically cooled holder with a thermistor for monitoring and adjusting the heat sink temperature. The optical power and spectra from front facets of the lasers were measured with a calibrated thermopile detector placed directly in front of the laser facets and a Fourier transform infrared (FTIR) spectrometer, respectively.

### Low Threshold Continuous Wave Operation

**Fig. 2:**
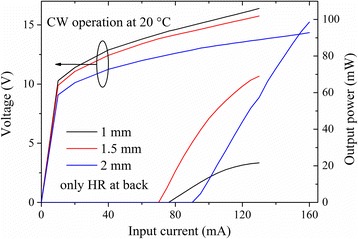
Typical CW *PIV* characterization of the DFB QCLs with different cavity lengths of 1, 1.5, and 2 mm and ridge width of 9 μm

Figure 2 shows the CW power-current-voltage (*PIV*) characterization of three DFB QCLs with the cavity lengths of 1, 1.5, and 2 mm and ridge width of 9 μm. These data were measured at the heat sink temperature of 20 °C, from the devices with HR coating on the back facet and no coating on the front facet. The operating voltage decreased as the cavity length increased. The threshold currents (*I*_th_) of 1-, 1.5-, and 2-mm-long devices were 76, 70, and 90 mA, respectively, with the corresponding *J*_th_ of 0.84, 0.52, and 0.5 kA/cm^2^. The maximum CW output power were 22, 69, and 99 mW for 1-, 1.5-, and 2-mm-long devices. It is noticed that the *J*_th_ of a 1-mm-long device was remarkably higher, which could be further improved by PHR coating.

### High Temperature Low Power-Consumption Performance

After the CW measurement above, an Al_2_O_3_/Ge coating consisting of 220-nm-thick Al_2_O_3_ and 240-nm-thick Ge was deposited on the front facet of the 1-mm-long DFB QCL. The 1-mm-long device with both HR and PHR coatings realized very high CW performance, including high temperature, low power-consumption, high output power, stable single-mode emission, and high slope efficiency.

**Fig. 3:**
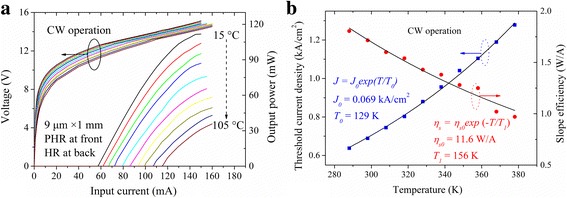
**a** CW *PIV* characterization of the 1-mm-long PHR and HR-coated DFB QCL and **b** the *J*_th_ and *η*_*s*_ as the functions of the heat sink temperature

Figure 3a shows the CW *PIV* characterization of the 1-mm-long device with both HR and PHR coatings over a heat sink temperature range from 15 to 105 °C. At 15 °C, the CW *I*_th_ and *J*_th_ were 57.4 mA and 0.64 kA/cm^2^, corresponding to the power consumption of 0.72 W. The maximum output power was more than 110 mW at the input electrical power-consumption of 2.2 W. As the heat sink temperature increased, the *I*_th_ increased to 115 mA and the maximum output power was still 36 mW at 105 °C. Limited by the heat sink temperature adjusting stage, we believe that the 1-mm-long device can operate well above 105 °C. The low-power consumption and high operating temperature mean that the cooling system for removing the heat can be simplified or even removed, which is significant to many applications. In addition, high temperature and low power-consumption performance was also important for applications in harsh thermal environments. The *J*_th_ and maximum slope efficiency (*η*_*s*_) were also plotted as the functions of the heat sink temperature in Fig. 3b. The *η*_*s*_ was 1.8 W/A at 15 °C and about 1.0 W/A at 105 °C, which is much higher than the previously reported *η*_*s*_ values [8–11]. The characteristic temperatures *T*_0_ and *T*_1_ for threshold current density and slope efficiency were 129 and 156 K, respectively. Both the low *J*_th_ and high *η*_*s*_ were excellent values for short cavity low power-consumption DFB QCLs, meaning high WPE in CW mode for the 1-mm-long device.

**Fig. 4:**
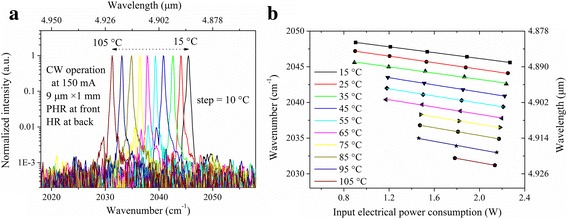
**a** CW lasing spectra of the 1-mm-long PHR and HR-coated DFB QCL at 150 mA with different heat sink temperatures and **b** CW lasing wavenumber as a function of input electrical power-consumption and heat sink temperature

Figure 4a shows the CW lasing spectra of the 1-mm-long PHR and HR-coated DFB QCL at 150 mA with the heat sink temperatures ranging from 15 to 105 °C. As shown, the single mode emission with the side-mode-suppression ratio (SMSR) above 20 dB was tuned from 2045.5 to 2031.2 cm^−1^ as the temperature increased from 15 to 105 °C. The CW lasing wavenumber was also plotted as a function of input electrical power-consumption and heat sink temperature in Fig. 4b. It can be observed that the lasing wavenumber was tuned linearly with the input electrical power-consumption and heat sink temperature over the whole measured range, which indicates that stable single mode operation was maintained without mode hopping behavior.

As shown, the performance of the 1-mm-long DFB QCL was improved significantly by the PHR coating. We expect to further reduce the power consumption of our DFB QCL by shortening the cavity length and using higher reflectivity PHR coating.

### High Wall-Plug Efficiency

**Fig. 5:**
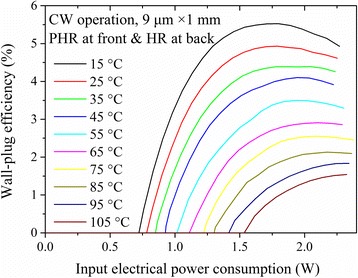
Wall-plug efficiency as a function of the electrical power dissipation for the 1-mm-long PHR and HR-coated DFB QCL

From Fig. 3a, the CW WPE was calculated and plotted as a function of the input electrical power-consumption in Fig. 5. At 15 °C, a maximum WPE of 5.5% was obtained around 125 mA with the output power of 100 mW. The maximum WPE were still 3.5 and 1.5% at 55 and 105 °C. To date, these values were very high for the DFB QCLs with the threshold power-consumption less than 1 W. It is believed that the WPE can be further improved by the precise facets coating technology and the optimized active region structure such as shallow-well design.

## Conclusions

Very low power-consumption DFB QCLs were fabricated by conventional process without lateral regrowth or using sidewall grating. The low *J*_th_ room temperature CW operation was demonstrated, which can be attributed to the optimized materials and low waveguide loss. The 1-mm-long PHR and HR-coated DFB QCL achieved very high CW performance, including high operating temperature (up to 105 °C), low threshold power-consumption (0.72 and 0.78 W at 15 and 25 °C), stable single-mode emission (SMSR >20 dB and mode-hopping free), high WPE of 5.5% and high slope efficiency of 1.8 W/A at 15 °C. The maximum CW output power was over 110 mW at 15 °C and still more than 35 mW at 105 °C. Compared with the previous reports on low power-consumption DFB QCLs, the fabrication process was simple and low cost. Meanwhile, the high temperature CW operation with low power-consumption allowed for more compact laser package, which is significant to develop portable laser system and for applications in harsh thermal environments.

## Abbreviations

BH: Buried heterostructure
CW: Continuous wave
DFB: Distributed feedback
EL: Electroluminescence
FTIR: Fourier transform infrared
FWHM: Full-width-at-half-maximum
HR: High reflection
*I*_th_: Threshold current
*J*_th_: Threshold current density
MBE: Molecular beam epitaxy
MOVPE: Metal organic vapor phase epitaxy
PHR: Partial-high-reflection
*PIV*: Power-current-voltage
QCL: Quantum cascade laser
*R*: Reflectivity
SMSR: Side-mode-suppression ratio
WPE: Wall-plug efficiency
*η*_*s*_: Slope efficiency
